# Preparation of ZnO Nanorods/Graphene Composite Anodes for High-Performance Lithium-Ion Batteries

**DOI:** 10.3390/nano8120966

**Published:** 2018-11-23

**Authors:** Junfan Zhang, Taizhe Tan, Yan Zhao, Ning Liu

**Affiliations:** 1 School of Materials Science and Engineering, Hebei University of Technology, Tianjin 300130, China; 18722593259@163.com; 2 Synergy Innovation Institute of GDUT, Heyuan 517000, China; tztansii18@163.com

**Keywords:** ZnO/graphene composites, anode, lithium-ion battery

## Abstract

ZnO is a promising anode material for lithium-ion batteries (LIBs); however, its practical application is hindered primarily by its large volume variation upon lithiation. To overcome this drawback, we synthesized ZnO/graphene composites using the combination of a simple hydrothermal reaction and spray drying. These composites consisted of well-dispersed ZnO nanorods anchored to graphene. The folded three-dimensional graphene spheres provided a high conductivity, high surface area, and abundant defects. LIB with an anode composed of our novel ZnO/graphene material demonstrated a high initial discharge capacity of 1583 mAh g^−1^ at 200 mA g^−1^.

## 1. Introduction

Recent ever-increasing demands for portable electronics (e.g., tablets, laptops, smart phones, etc.) have stimulated the research and development of lithium ion batteries (LIBs) due to their higher gravimetric capacity, longer lasting stability and lack of memory effects [1,2,3]. The theoretical capacity of the existing commercial graphite anodes of 372 mAh/g is unsatisfactory and barely meets the threshold of the required energy density [4]. Metal oxides, such as SnO_2_ [5], Fe_3_O_4_ [6], MnO_2_ [7], ZnO [8], etc., have attracted a lot of attention as alternative electrode materials because of their high gravimetric capacity. Among them, ZnO possesses many advantages including its high theoretical capacity, low cost, convenient preparation methods, minimal pollution and exceptional electrochemical performance. However, ZnO is rarely applied in LIBs due to its poor cycling performance [9,10,11], limited electrical conductivity and huge volumetric change during its charging and discharging processes [12]. To overcome these drawbacks, nano-ZnO [13], metal-modified ZnO particles [14] and ZnO/carbons composites [15,16] were fabricated. The impact of the ZnO nanoparticles’ morphology on their electrochemical performance was explored by producing anodes based on ZnO nanoplates, nanotubes, nanorods and nanospheres, all of which demonstrated significantly improved electrochemical performance compared to their bulk counterparts because of the high electrode stability and large surface area of the active material. However, low conductivity and serious volume change during ion intercalation/deintercalation still hindered even nano-ZnO applications as anodes. Anodes based on nano-ZnO often demonstrated insufficient rate capability and serious capacity degradation upon cycling [17,18,19,20]. 

Carbon materials have high electronic conductivity and negligible volume change. In particular, graphene is a very appealing three-dimensional (3D) substrate for various metal oxides [21,22] because of its large surface area and abundant defects, which could efficiently limit nano-ZnO diffusion and aggregation. Graphene can also withstand the volume changes associated with the lithiation/delithiation without much damage to its structure-enhancing and stabilizing electrochemical performance of the electrode. These advantages of graphene were reported in many studies [23,24,25,26] and a reversible capacity as high as 516 mAh g^−1^ after 100 cycles at 200 mA g^−1^ current density was obtained for anodes consisting of ZnO/graphene composites [1].

Inspired by these results, we also synthesized a high performance ZnO/graphene composite using the combination of a simple hydrothermal reaction and spray drying. Different from that previously reported (10.1039/c7se00102a), we use the spray-drying method to prepare a ZnO/graphene composite which not only can uniformly mix the raw materials but also causes the mixture to form granules. Graphene in the electrode materials forms a three-dimensional conductive network, which will facilitate electron migration. The initial discharge capacity of the anode composed of ZnO/graphene composite as an active material was 1583 mAh g^−1^ at 200 mA g^−1^ current density. After 100 cycles, the discharge capacity was as high as 886 mAh g^−1^.

## 2. Materials and Methods 

First, 0.8 g of sodium hydroxide (NaOH) and 0.2 g of zinc acetate ((CH_3_COO)_2_Zn·2H_2_O) were dissolved in 40 mL of deionized water, stirred for 2 h, then transferred into a 50 mL stainless steel vessel and heated at 140 °C for 2 h. Finally, the resultant ZnO were collected and washed by deionized water five times and then dried under vacuum overnight.

The prepared ZnO/graphene composite was spray-dried by a spray-drier system (HOLVES). First of all, graphene oxide (GO) was prepared according to Hummers’ method and diluted by distilled water to obtain a GO suspension with a concentration of 2 mg mL^−1^. Then, 0.4 g ZnO was dispersed in 100 mL of GO suspension and stirred for 5 h. Subsequently, the ZnO/GO suspension was further dried via a spray dryer to produce the composite powder. Finally, the obtained black powders were collected and further reduced to ZnO/graphene by using hydrazine hydrate. A reference pure ZnO nanorod without graphene was prepared following the same conditions.

Composites were tested by X-ray diffraction (XRD, D8 Discover, Bruker, Billerica, MA, USA) from 10° to 80° of 2θ at 12 °/min. Scanning electron microscopy (SEM, Hitachi Limited, Tokyo, Japan) was implemented using Hitachi S-4800. Transmission electron microscopy (TEM, JEOL, Tokyo, Japan) images were obtained using a JEOL-2100F operated at 200 kV. Raman spectra and X-ray photoelectron spectroscopy were recorded by Lab RAM HR800 (HORIBA Jobin Yvon, Tokyo, Japan) and Thermo Scientific K-Alpha XPS spectrometer (XPS, Thermo Scientific, Massachusetts, USA), respectively. The thermogravimetric analysis (TGA, TA Instruments-Waters LLC, Newcastle, PA, USA) was carried out under oxygen using a TGA Q600 system. Cyclic voltammogram (CV) curves were performed by an electrochemical workstation (Princeton, Versa STAT 4, Ametek, PA, USA) in a voltage range of 0.01–3.00 V.

Then, 80 wt% ZnO/graphene composite, 10 wt% Ketchen black and 10 wt% polyvinylidene fluoride (PVDF) were mixed in 1-methyl-2-pyrrolidinone (NMP) to form a slurry and applied onto the Cu foil by a draw-down blade. After drying at 60 °C for 12 h, 9 mm disks were punched that served as anodes. The ZnO/graphene loading amount of each electrode sheet was around 2 mg cm^−2^. Coin-type (CR2032) half-cells were assembled in an argon-filled glove box using these anodes as well as lithium foil as a counter electrode and Celgard 2300 as a separator. The electrolyte was 1.0 M LiPF_6_/ (EC + DMC + DEC + EMC) (with the 1:1:1:3 ratio by volume). The galvanostatic charge–discharge tests were conducted using Neware battery tester (Shenzhen, China) in the 0.01–3.0 V voltage window (vs. Li^+^/Li).

## 3. Results and Discussion

**Figure 1:**
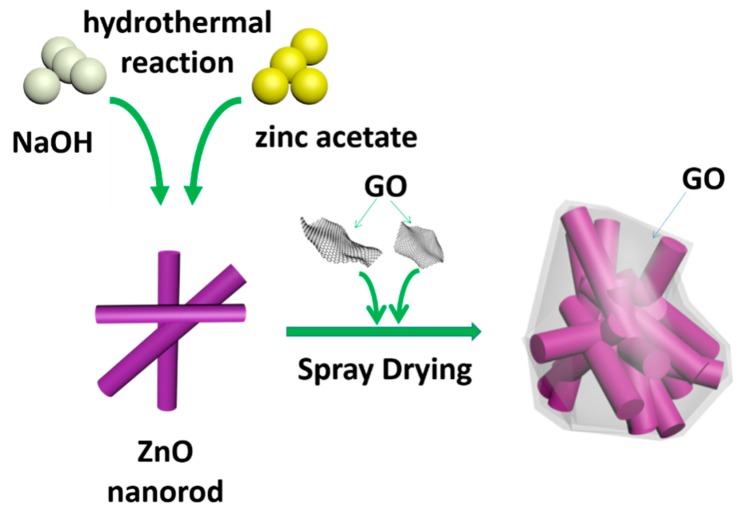
Schematic illustration of the synthesis of the ZnO/graphene composite.

The preparation process and structure diagram of of ZnO/graphene composite is depicted in Figure 1.

**Figure 2:**
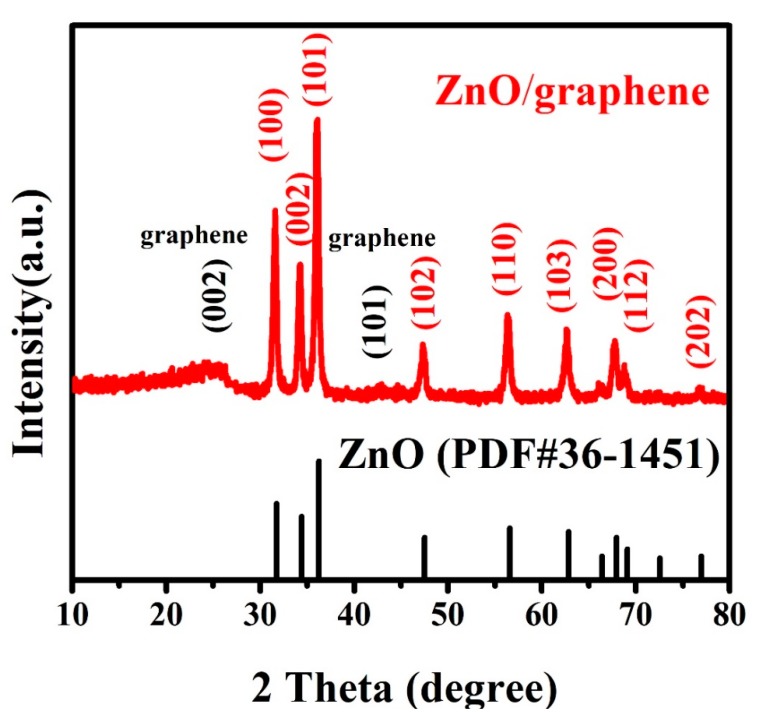
XRD pattern of the ZnO/graphene composites (top) in comparison with the XRD peaks of the pure ZnO from PDF #36-1451 (bottom).

All XRD peaks matched to the pattern of hexagonal ZnO (PDF#36-1451) (as shown in Figure 2). Peaks at ~31.8°, 34.4°, 36.4°, 47.5°, 56.6°, 62.8°, 67.8°, 69.1° and 76.9° correspond to the (100), (002), (101), (102), (110), (103), (200), (112) and (202) crystal planes of ZnO, respectively. No XRD peaks of graphene oxide (2θ = 10°) were observed, and two major diffraction peaks located at 2θ = 26.4° and 44.4° were observed, which correspond to the (002) and (101) crystal planes of graphene, indicating that GO has been converted to RGO.

**Figure 3:**
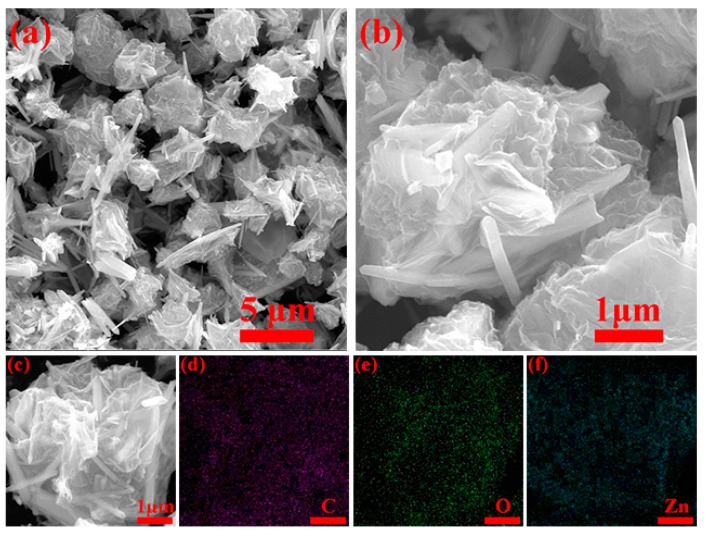
SEM micrographs of (**a**–**c**) ZnO/graphene composites and (**d**–**f**) corresponding EDS mapping of (**c**).

**Figure 4:**
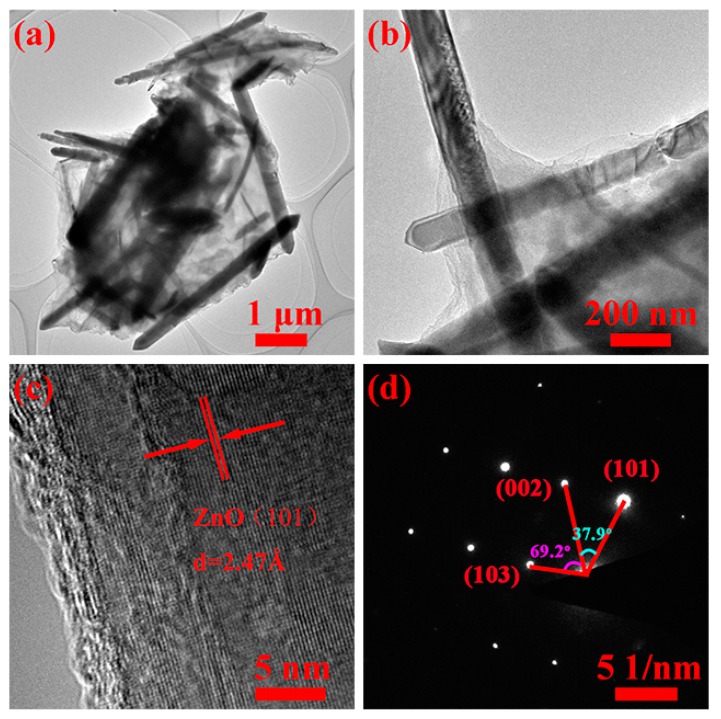
TEM images of (**a**,**b**) ZnO/graphene composites and (**c**) a single ZnO nanoparticle; (**d**) selected area electron diffraction (SAED) pattern of ZnO.

SEM showed 2–5 μm size spherical ZnO/graphene composites (see Figure 3a,b). Both SEM and TEM (shown in Figure 4a,b) images demonstrate that ZnO nanorods overlap with each other and are tightly wrapped in graphene spheres. We believe that such a composite structure should have a significant mitigation effect on the ZnO volume expansion/contraction during the charge/discharge processes. Graphene also provides fast transfer channel for electrons, which is extremely important for efficient electrochemical performance at high currents. Elemental mappings of the composites (see Figure 3c) showed uniform distribution of carbon, oxygen and zinc (Figure 3d–f) indicating that some ZnO nanorods were completely encapsulated inside the graphene. High-resolution TEM (shown in Figure 3c) clearly indicates the (101) crystal plane of ZnO with interplanar distance equal to ~2.47 Å, and SAED shows the (103), (002) and (101) crystal planes of hexagonal ZnO (see Figure 4d).

**Figure 5:**
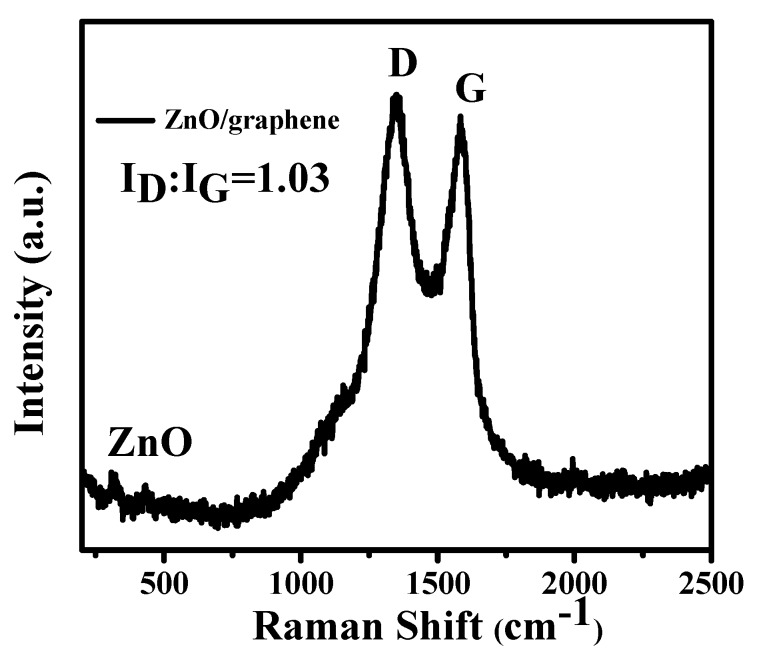
Raman spectrum of ZnO/graphene composite.

Raman spectroscopy of the ZnO/graphene composite showed two peaks at ~1346 and 1581 cm^−1^ (see Figure 5), which belong to the D and G bands of graphene. The intensity ratio of the D and G bands (I_D_/I_G_) is 1.03, indicating a high degree of defects of graphene in the composite, which can facilitate Li^+^ transportation during charge and discharge cycling. Weak peaks between 250 and 500 cm^−1^ can be attributed to ZnO.

**Figure 6:**
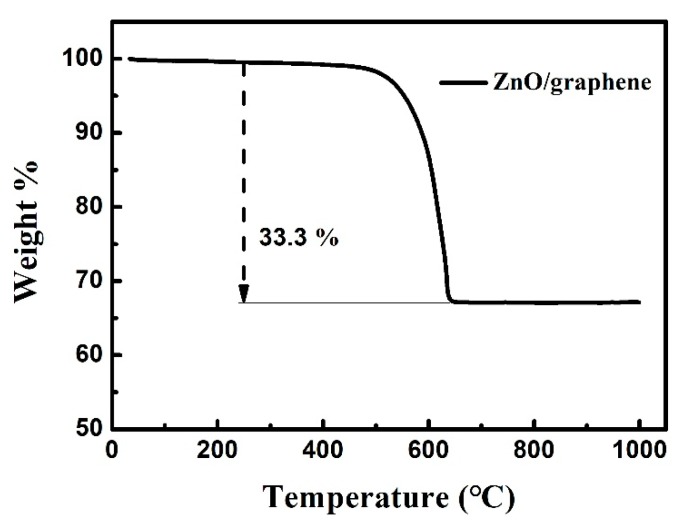
Thermogravimetric analysis (TGA) analysis of the ZnO/graphene composite.

In order to investigate the quality of the graphene in the ZnO/graphene composite, the thermogravimetric analysis (TGA) of the ZnO/graphene was performed under oxygen (see Figure 6). The weight loss between 480 °C and 620 °C is mainly a result of the removal of graphene sheets, indicating that the quality of the graphene is about 33.3%.

**Figure 7:**
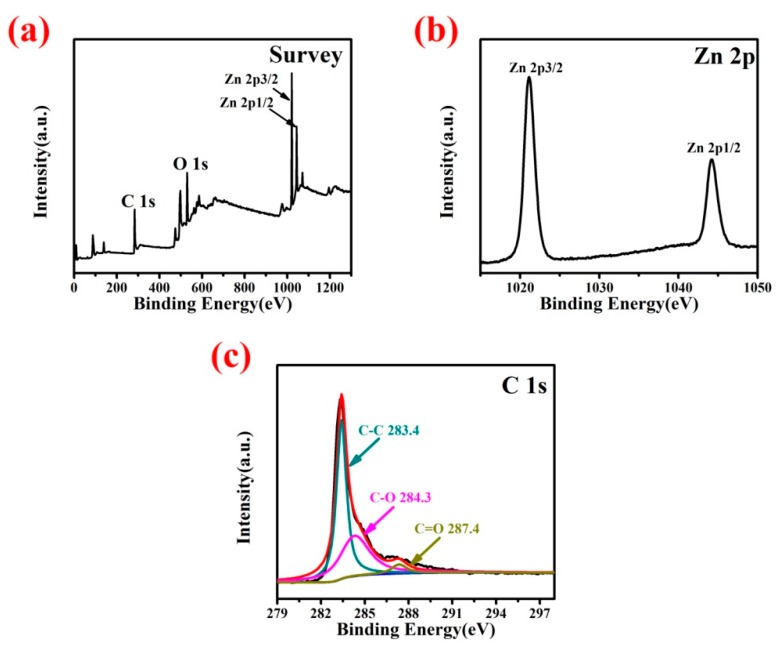
(**a**) Survey, (**b**) Zn 2p and (**c**) C 1s spectra of ZnO/graphene composites.

XPS showed only Zn, O and C, confirming the high purity of the synthesized ZnO/graphene composites. Peaks at 1021.2 and 1044.1 eV can be attributed to Zn 2p 3/2 and Zn 2p 1/2, respectively. Peaks corresponding to the graphene functional groups were observed at 283.4 eV for the C-C bond, 284.3 eV for C-O bond and 287.4 eV for C=O bond (see Figure 7c). Intensities of the peaks associated with the carbon–oxygen functional groups were much lower than the intensities of carbon–carbon bonds. Thus, graphene oxide was mostly reduced after the hydrothermal reaction and spray drying.

**Figure 8:**
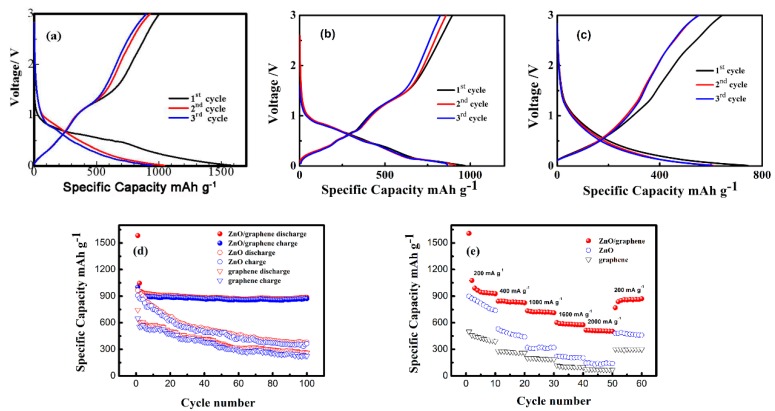
(**a**–**c**) Charge–discharge curves of the ZnO/graphene, ZnO and graphene. (**d**) Cycle performance of the ZnO/graphene, ZnO and graphene anodes at 200 mA g^−1^. (**e**) Rate capability of the ZnO/graphene, ZnO and graphene anodes.

Figure 8a–c shows the first three-cycle performances of the ZnO/graphene, pure ZnO and graphene at a current density of 200 mA g^−1^. The discharge curve for the first cycle of the ZnO/graphene electrodes at 200 mA g^−1^ showed a plateau at 0.5–0.7 V, which can be linked to the reduction of ZnO into Zn. A platform at ~1.2 V on the corresponding charge curve was because of the reappearance of ZnO from Li–Zn alloy and Zn [27]. The excellent electrochemical capacity of the ZnO/graphene composites anodes was also attributed to the graphene in the composites; this can not only keep the electrode structure from collapsing, but also provides the electronic conduction during the charging and discharging processes and provides capacity at the electrode. The first discharge and charge capacities were 1583 and 1002 mAh g^−1^, respectively. Such a big difference is very likely due to the solid electrolyte interphase (SEI) formation as well as to the incomplete Zn oxidation relative to the initial ZnO amount. Charge and discharge capacities for the second cycle of the ZnO/graphene anode were the same, demonstrating good electrode stability. Compared with the ZnO/graphene composite, both the pure ZnO and graphene revealed low initial capacity (see Figure 8b,c), which is consistent with cycling performance (see Figure 8d)

The outstanding stability of our ZnO/graphene anode was further confirmed by its cycling performance, shown in Figure 8d. The specific discharge capacity decreased from 1045 to 886 mAh g^−1^ after 100 cycles. The capacity attenuation rate was 15.2% and the capacity attenuation per cycle was only 0.15%. Remarkably, compared to the ZnO/graphene, both the ZnO and graphene anodes show a significant decrease in charge and discharge capacity. The presence of graphene coating mitigated the volume change during the charge/discharge processes [28,29]. The conductivity of the electrode was also improved so that the electron transport was accelerated. The synergy between ZnO and graphene improved the cycle stability of the cell, utilizing maximum capacity of ZnO.

Our rate capability tests showed discharge capacities equal to 938, 835, 718, 582 and 508 mAh g^−1^ at 200, 400, 1000,1600, 2000 mA g^−1^ current density, respectively (see Figure 8e). After the cycling at the highest rate (2000 mA g^−1^), the discharge capacity increased to 859 mAh g^−1^ when cycling was performed again at 200 mA g^−1^. It can be observed that the ZnO/graphene anodes have a better rate of performance than pure ZnO and pure graphene anodes, indicating the good rate capability of the ZnO/graphene anode.

**Figure 9:**
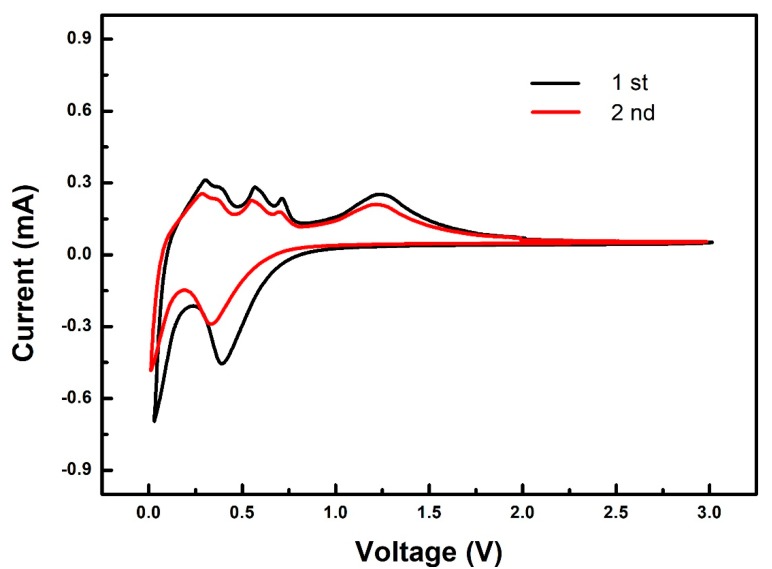
Cyclic voltammogram (CV) curves of the ZnO/graphene composite electrode at a scan rate of 0.1 mV s^−1^.

Figure 9 shows the CV curves of the ZnO/graphene composite electrode at a scan rate of 0.1 mV s^−1^. Decomposition of the electrolyte and formation of the solid electrolyte interface on the surface of electrode particles also occurs during the first cathodic cycle, which causes part of the irreversible capacity [17]. During the anodic scanning process, anodic peaks located at 0.27, 0.36, 0.57, and 0.72 V are observed, which is attributed to the multi-step de-alloying process of Li-Zn (LiZn, Li_2_Zn_3_, LiZn_2_, and Li_2_Zn_5_). Another bigger anodic peak at 1.26 V is attributed to the back-conversion of Zn and Li into ZnO [9].

## 4. Conclusions

ZnO/graphene composites were prepared by the combination of a simple hydrothermal reaction and spray drying. ZnO was prepared by a hydrothermal reaction and ZnO/graphene composites 2–5 μm in diameter were achieved during the second step of spray drying. ZnO nanorods in the composite overlapped with each other and were tightly wrapped in graphene. Graphene enhanced the conductivity of the composite electrode and served as a buffer for the volume change. The synergistic effect of graphene and ZnO gave the cell excellent cycle stability. The results illustrated that micro-spherical ZnO/graphene composite material is an excellent candidate as an anode material for LIBs.
